# Use of Pleural Fluid Digital PCR Analysis to Improve the Diagnosis of Pleural Tuberculosis

**DOI:** 10.1128/spectrum.01632-22

**Published:** 2022-10-20

**Authors:** Zihui Li, Qi Sun, Boping Du, Hongyan Jia, Jing Dong, Lingna Lyu, Chuanzhi Zhu, Aiying Xing, Xinting Yang, Rongrong Wei, Xiaoyou Chen, Zongde Zhang, Liping Pan

**Affiliations:** a Beijing Key Laboratory for Drug Resistant Tuberculosis Research, Beijing Chest Hospital, Capital Medical University, Beijing Tuberculosis and Thoracic Tumor Research Institute, Beijing, China; b Beijing Ditan Hospital, Capital Medical University, Beijing, China; Shenzhen University School of Medicine

**Keywords:** tuberculosis, pleural tuberculosis, digital PCR, pleural fluid, pleural effusion, diagnosis

## Abstract

The diagnosis of pleural tuberculosis (TB) remains difficult due to the paucity of Mycobacterium tuberculosis in pleural fluid (PF). This study aimed to improve pleural TB diagnosis using highly sensitive digital PCR (dPCR) technique. A total of 310 patients with evidence of PF were consecutively enrolled, 183 of whom suffered from pleural TB and 127 from non-TB. PF samples were prospectively collected and total DNA was extracted. The copy numbers of M. tuberculosis insertion sequence (IS) *6110* and IS*1081* in DNA were quantified using dPCR. The overall area under the curve of IS*6110-*dPCR was greater than that of IS*1081-*dPCR (0.85 versus 0.79). PF IS*6110* OR IS*1081-*dPCR (according to their cut-off values, “positive” was defined as either of them was positive, while “negative” was defined as both of them were negative) had higher sensitivity and equal specificity compared with single target-dPCR. The sensitivity of PF IS*6110* OR IS*1081-*dPCR for total, definite, and probable pleural TB was 59.0% (95% CI = 51.5% to 66.2%), 72.8% (95% CI = 62.6% to 81.6%), and 45.1% (95% CI = 34.6% to 55.8%), respectively. Its specificity was 100% (95% CI = 97.1% to 100.0%). PF IS*6110* OR IS*1081-*dPCR showed a higher sensitivity than smear microscopy (57.4% versus 7.1%), mycobacterial culture (55.3% versus 31.8%), and Xpert MTB/RIF (57.6% versus 23.0%). Long antituberculosis treatment time (>1 month) was found to be associated with negative dPCR results in pleural TB patients. This study indicates that PF IS*6110* OR IS*1081*-dPCR is an accurate molecular assay, which is more sensitive than routine etiological tests and has the potential to enhance the definite diagnosis of pleural TB.

**IMPORTANCE** Pleural TB is one of the most frequent causes of pleural effusion, especially in areas with high burden of TB. Due to the paucibacillary nature of the disease, the diagnostic sensitivities of all available bacteriological and molecular tests remain poor. There is an urgent need to develop new efficient methods. Digital PCR (dPCR) is the third generation of PCR that enables the exact quantification of trace nucleic acids in samples. This study evaluates the diagnostic performance of pleural fluid (PF) dPCR analysis for pleural TB, and shows that PF IS*6110* OR IS*1081-*dPCR has a higher sensitivity than routine etiological tests such as smear microscopy, mycobacterial culture, and Xpert MTB/RIF. This work provides a new choice for improving the definite diagnosis of pleural TB.

## INTRODUCTION

Tuberculosis (TB) was the leading cause of death from a single infectious agent until the COVID-19 pandemic. Globally in 2020, an estimated 9.87 million people fell ill with TB, and 1.51 million people died from the disease (1). Pleural TB is a common type of TB with an incidence of 3% to 5% in nonendemic areas and 30% in endemic areas with a high proportion of HIV-positive individuals (2). Pleural thickening, tuberculous empyema, chylothorax, and pneumothorax are possible complications of pleural TB, which can cause lung function impairment, chronic chest pain, or dyspnea, and bring great harm to patients (3–5).

Rapid diagnosis and timely treatment can reduce the risk of severe complications. However, the diagnosis of pleural TB is quite challenging. Due to the paucity of Mycobacterium tuberculosis in pleural fluid (PF), current microbiological and molecular tests for definite diagnosis of pleural TB show poor sensitivities (direct PF smear microscopy with Ziehl-Nielsen or Auramine staining < 10%, mycobacterial culture usually < 30%, automated nucleic acid amplification tests [NAAT] Xpert MTB/RIF [Xpert] [Cepheid, Sunnyvale, CA, USA] 21.4% to 22.7% and its next-generation Xpert Ultra 37.5% to 48.2%) (2, 6–13). Pleural biopsy combined with the above methods is more sensitive, but it is invasive and not suitable for resource-limited settings (14, 15). Other biochemical parameters (such as adenosine deaminase [ADA] and interferon gamma [IFN-γ]) or immunological methods (such as tuberculin skin test [TST] and IFN-γ release assays [IGRAs]) have poor diagnostic accuracy for pleural TB (16–19). Therefore, new efficient methods are urgently needed to improve pleural TB diagnosis.

Digital PCR (dPCR) is a powerful method for the absolute quantification of low-abundance nucleic acids. Compared with real-time quantitative PCR (qPCR), dPCR has greater precision, better reproducibility, higher tolerance to inhibitors, and does not rely on calibration curves for quantification (20–25). In recent years, dPCR has been utilized in gene expression analysis, pathogen detection, epigenetic analysis, rare mutation detection, copy number variation analysis, noninvasive prenatal testing, etc. (24–30). Whether it can be used to improve the diagnosis of pleural TB has not been reported. In this study, we aimed to evaluate the diagnostic performance of PF dPCR analysis for pleural TB.

## RESULTS

### Characteristics of participants.

A total of 310 patients with pleural effusion were enrolled; 183 of whom were pleural TB patients (including 92 definite cases and 91 probable cases) and 127 were non-TB patients. Eighty-eight percent (161/183) of pleural TB patients were accompanied by pulmonary or other organ TB. Among non-TB patients, 12 were diagnosed as malignant pleural mesothelioma, 92 as lung cancer with pleural metastasis (including four squamous carcinoma, 77 adenocarcinoma, and 11 small cell carcinoma), 23 as other cancers with pleural metastasis (including five breast cancer, one ovarian cancer, one thymoma, one esophageal cancer, one Ewing's sarcoma, and one non-Hodgkin's lymphoma, and 13 as malignant pleural effusion with unknown primary site). Overall, 215 patients (69.4%) were male and the median age was 55 years old (range from 16 to 93). Pleural TB patients were younger than non-TB patients and had a higher proportion of males. Baseline characteristics of the study population are presented in Table 1.

**TABLE 1:** Basic demographic and clinical characteristics of study participants (*n* = 310)

Characteristics	Definite pleural TB	Probable pleural TB	Total pleural TB	Non-TB	Total patients	*P* value^*a*^
Patients no.	92	91	183	127	310	
Age, median (range), yr	41 (16 to 85)	51 (17 to 93)	47 (16 to 93)	64 (24 to 88)	55 (16 to 93)	< 0.0001
Gender(male/female)	78/14	71/20	149/34	66/61	215/95	< 0.0001
No. of patients combined with pulmonary or other organ TB	70	91	161	-^*b*^	-	-
Antituberculosis treatment time						
≤1 mo	85	82	167	-	-	-
>1 mo	7	9	16	-	-	-

a Comparison between total pleural TB and non-TB.

b -, not applicable.

### Performance of IS*6110-* and IS*1081*-targeted dPCR.

**FIG 1:**
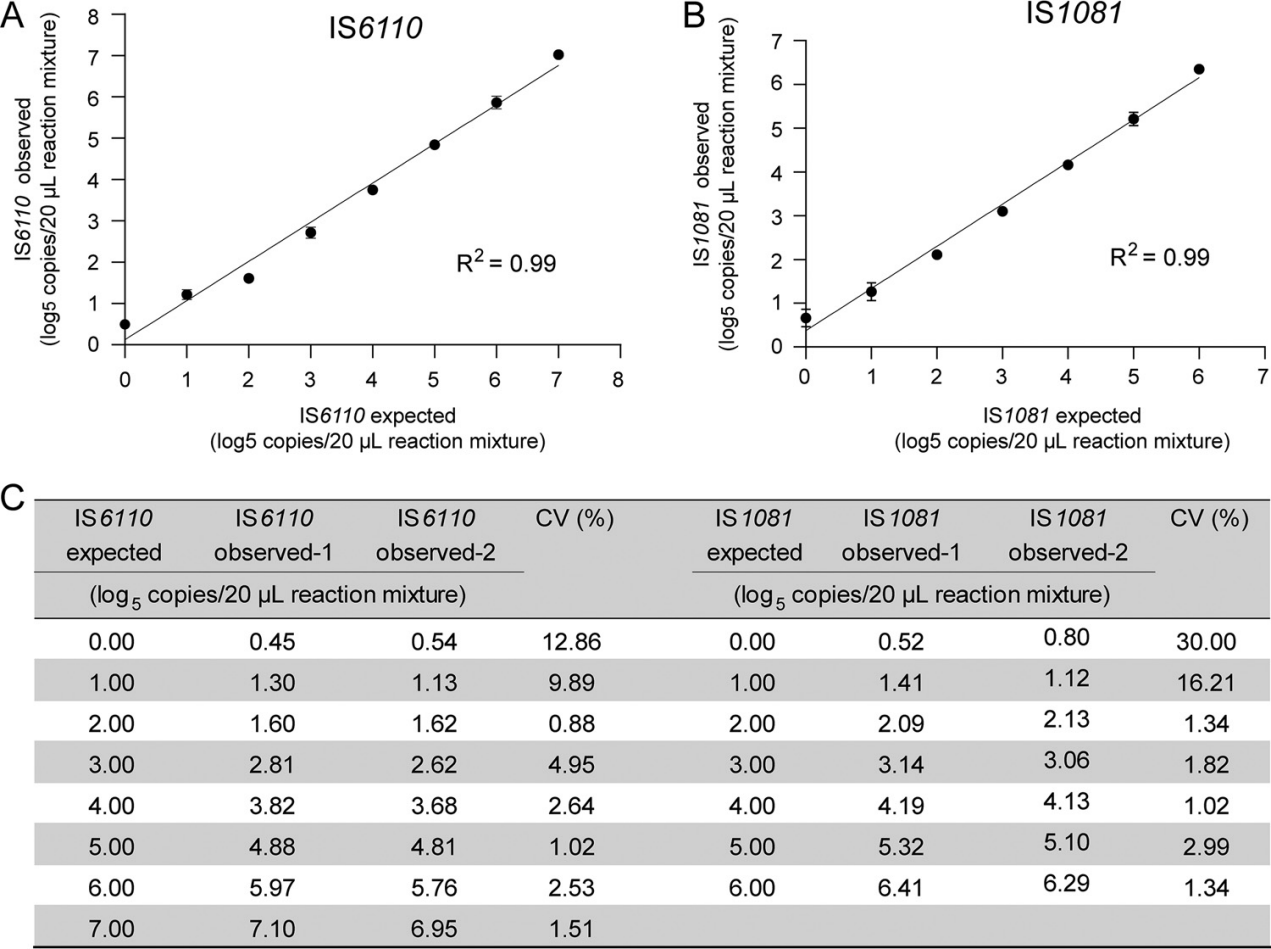
Performance of IS*6110*- and IS*1081*-targeted dPCR. Linear correlations between the observed and the expected IS*6110* (A) and IS*1081* (B) load, expressed as log_5_ copies/20-μL reaction mixture. Bars represent mean (±SD). Reproducibility analysis confirmed the high reliability of the methods (C). Each value was tested in two independent experiments, each led in triplicate.

IS*6110-* and IS*1081*-targeted dPCR exhibited excellent linear correlation between observed and expected targets quantification in an observed dynamic range of approximately 2.3 to 82,067 (IS*6110*, R^2^ = 0.99) and 3.0 to 27,520 (IS*1081*, R^2^ = 0.99) copies/20-μL PCR mixture (Fig. 1A and B). Repeatability analysis confirmed the high reliability of the method and the detailed coefficient of variation (CV) values of the two experiments are listed in Fig. 1C.

### Results of dPCR in detection of M. tuberculosis nucleic acids in PF.

**FIG 2:**
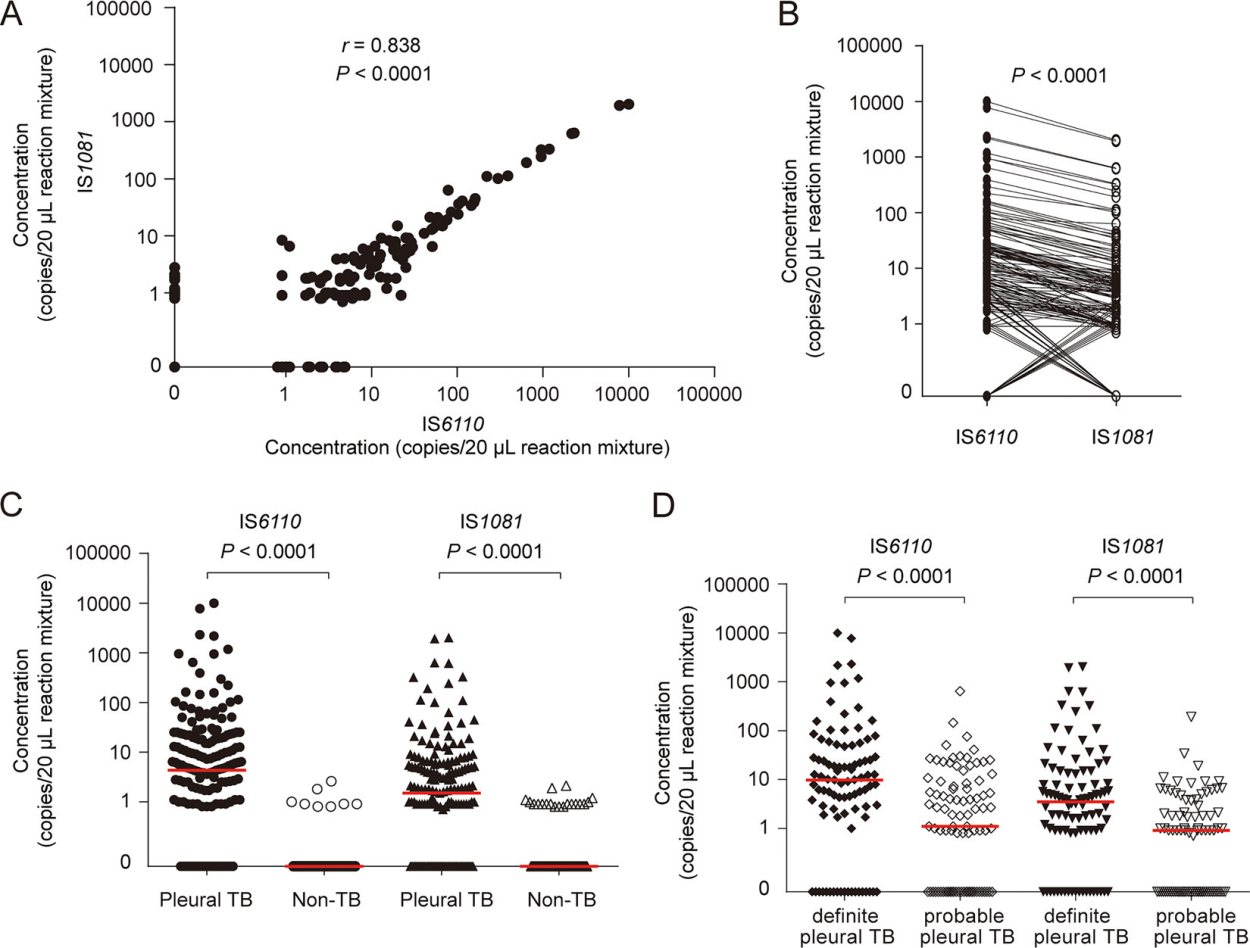
Quantification of M. tuberculosis DNA in pleural fluid samples by dPCR. (A) and (B) showed the correlation (Spearman correlation test) and the differences (Wilcoxon test) in the number of copies detected between IS*6110*- and IS*1081*-dPCR, respectively. (C) IS*6110* and IS*1081* copies detected in pleural TB and non-TB group, respectively (Mann-Whitney *U* test). (D) IS*6110* and IS*1081* copies detected in definite and probable pleural TB patients, respectively (Mann-Whitney *U* test). The number of copies per 20-μL reaction mixture was calculated as the average of the results in two independent experiments, each led in duplicate. Results were considered significant when *P* < 0.05.

IS*6110-* and IS*1081*-targeted tests were highly correlated with each other (*r *= 0.838, *P* < 0.0001, Fig. 2A), and the number of IS*6110* detected in the same sample was usually more than that of IS*1081* (*P* < 0.0001, Fig. 2B). The number of copies detected in pleural TB group was significantly higher than that in non-TB group: median (minimum, maximum), IS*6110*, 4.3 (0.0, 9990.0) *versus* 0.0 (0.0, 2.6) copies/20-μL reaction mixture, *P* < 0.0001; IS*1081*, 1.5 (0.0, 2020.0) versus 0.0 (0.0, 2.1) copies/20-μL reaction mixture, *P* < 0.0001 (Fig. 2C). The number of copies detected in definite pleural TB group was higher than that in probable pleural TB group: median (minimum, maximum), IS*6110*, 9.9 (0.0, 9990.0) versus 1.1 (0.0, 641.0) copies/20-μL reaction mixture, *P* < 0.0001; IS*1081*, 3.5 (0.0, 2020.0) versus 0.9 (0.0, 191.0) copies/20-μL reaction mixture, *P* < 0.0001 (Fig. 2D). Some original results are shown in Fig. S1.

### Performance of dPCR in pleural TB diagnosis.

**FIG 3:**
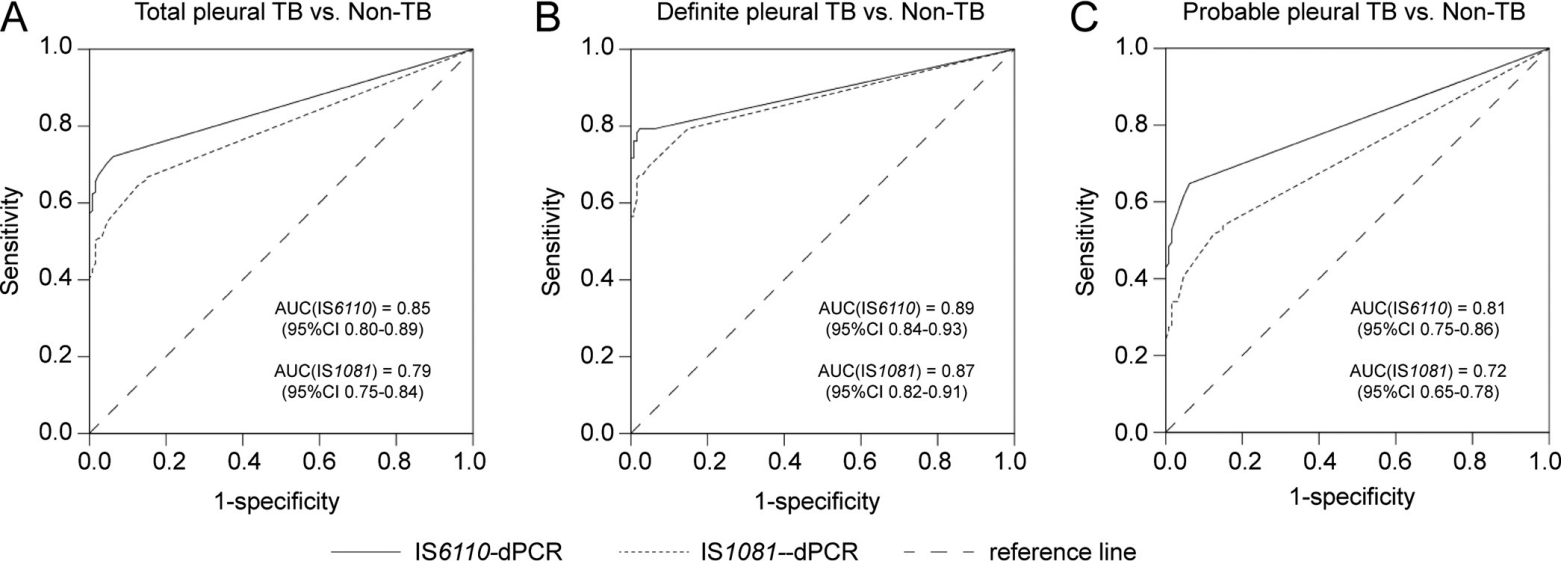
Diagnostic performances of pleural fluid dPCR assays for pleural TB. (A), (B), and (C) were receiver operating characteristic (ROC) curves in IS*6110*- and IS*1081*-dPCR for the diagnosis of total, definite, and probable pleural TB, respectively. AUC, area under ROC curve; CI, confidence interval.

The ability to detect M. tuberculosis DNA in pleural effusion was assessed with a receiver operating characteristic (ROC) analysis. The overall area under receiver operating characteristic curve (AUC) of IS*6110-*dPCR (0.85, 95% CI = 0.80 to 0.89) was larger than that of IS*1081-*dPCR (0.79, 95% CI = 0.75 to 0.84) (*P* = 0.002, Fig. 3A). The AUC of dPCR in patients with definite pleural TB was larger than that in patients with probable pleural TB (Fig. 3B and 2C).

The diagnostic performance of dPCR assay for pleural TB is presented in Table 2. The cut-off value was determined according to two principles: (i) ensure a high specificity (to be used as a rule-in test); and (ii) ensure that the sum of sensitivity and specificity is maximized under the premise of high specificity (to obtain the best diagnostic ability). In this study, 2.6 (IS*6110*) and 2.1 (IS*1081*) copies per 20-μL reaction mixture were defined as the optimal cut-off values to ensure high specificity and then the largest sum of sensitivity and specificity. The sensitivity of IS*6110-*dPCR assay for total pleural TB was higher than that of IS*1081*-dPCR assay (57.4% versus 40.4%, *P* < 0.001), and they had the same specificity (100%). The sensitivity of dPCR assay in definite pleural TB patients (IS*6110*, 71.7%; IS*1081*, 56.5%) was higher than that in probable pleural TB patients (IS*6110*, 42.9%; IS*1081*, 24.2%). The sensitivity of combined detection of IS*6110* and IS*1081* using dPCR (according to their cut-off values, the positive result was defined as either of them was positive, while the negative result was defined as both of them were negative; hereinafter referred to as IS*6110* OR IS*1081-*dPCR) for total, definite, and probable pleural TB was 59.0%, 72.8%, and 45.1%, respectively, and the specificity remained 100%.

**TABLE 2:** Diagnostic performances of pleural fluid dPCR assay for pleural TB

Tests	AUC^*d*^ (95% CI)^*i*^	Criterion (copies/20 μL reaction mixture)	Sensitivity (%) (95% CI)	Specificity (%) (95% CI)	LR+^*e*^	LR–^*f*^ (95% CI)	PPV^*g*^ (%) (95% CI)	NPV^*h*^ (%) (95% CI)
Total pleural TB (*n* = 183) compared with non-TB (*n* = 127)								
IS*6110*-dPCR	0.85 (0.80 to 0.89)	>2.6	57.4^*a*^ (49.9 to 64.6)	100.0 (97.1 to 100.0)	-	0.4 (0.4 to 0.5)	100.0 (96.5 to 100.0)	62.0 (54.9 to 68.6)
IS*1081*-dPCR	0.79 (0.75 to 0.84)	>2.1	40.4^*a*^ (33.3 to 47.9)	100.0 (97.1 to 100.0)	-	0.6 (0.5 to 0.7)	100.0 (95.1 to 100.0)	53.8 (47.2 to 60.3)
IS*6110* & IS*1081-*dPCR positive	0.69 (0.64 to 0.75)	>2.6 and >2.1	38.8 (31.7 to 46.3)	100.0 (97.1 to 100.0)	-	0.6 (0.5 to 0.7)	100.0 (94.9 to 100.0)	53.1 (46.6 to 59.6)
IS*6110* OR IS*1081-*dPCR positive	0.80 (0.75 to 0.84)	>2.6 or >2.1	59.0 (51.5 to 66.2)	100.0 (97.1 to 100.0)	-	0.4 (0.3 to 0.5)	100.0 (96.6 to 100.0)	62.9 (55.8 to 69.5)
Definite pleural TB (*n* = 92) compared with non-TB (*n* = 127)								
IS*6110*-dPCR	0.89 (0.84 to 0.93)	>2.6	71.7^*b*^ (61.4 to 80.6)	100.0 (97.1 to 100.0)	-	0.28 (0.2 to 0.4)	100.0 (94.6 to 100.0)	83.0 (76.1 to 88.6)
IS*1081*-dPCR	0.87 (0.82 to 0.91)	>2.1	56.5^*c*^ (45.8 to 66.8)	100.0 (97.1 to 100.0)	-	0.4 (0.3 to 0.5)	100.0 (93.0 to 100.0)	76.0 (68.8 to 82.3)
IS*6110* & IS*1081-*dPCR positive	0.78 (0.72 to 0.83)	>2.6 and >2.1	55.4 (44.7 to 65.8)	100.0 (97.1 to 100.0)	-	0.5 (0.4 to 0.6)	100.0 (93.0 to 100.0)	75.6 (68.4 to 81.9)
IS*6110* OR IS*1081-*dPCR positive	0.86 (0.81 to 0.91)	>2.6 or >2.1	72.8 (62.6 to 81.6)	100.0 (97.1 to 100.0)	-	0.3 (0.2 to 0.4)	100.0 (94.6 to 100.0)	83.6 (76.7 to 89.1)
Probable pleural TB (*n* = 91) compared with non-TB (*n* = 127)								
IS*6110*-dPCR	0.81 (0.75 to 0.86)	>2.6	42.9^*b*^ (32.5 to 53.7)	100.0 (97.1 to 100.0)	-	0.6 (0.5 to 0.7)	100.0 (90.7 to 100.0)	70.9 (63.7 to 77.5)
IS*1081*-dPCR	0.72 (0.65 to 0.78)	>2.1	24.2^*c*^ (15.8 to 34.3)	100.0 (97.1 to 100.0)	-	0.8 (0.7 to 0.9)	100.0 (84.6 to 100.0)	64.8 (57.7 to 71.5)
IS*6110* & IS*1081-*dPCR positive	0.61 (0.54 to 0.68)	>2.6 and >2.1	22.0 (14.0 to 31.9)	100.0 (97.1 to 100.0)	-	0.8 (0.7 to 0.9)	100.0 (82.4 to 100.0)	64.1 (57.0 to 70.8)
IS*6110* OR IS*1081-*dPCR positive	0.73 (0.66 to 0.78)	>2.6 or >2.1	45.1 (34.6 to 55.8)	100.0 (97.1 to 100.0)	-	0.6 (0.5 to 0.7)	100.0 (91.4 to 100.0)	71.8 (64.5 to 78.3)

a Sensitivity comparison between IS*6110-* and IS*1081*-dPCR in total pleural TB patients under the same specificity 100.0% (*P* = 0.000, McNemar test).

b Sensitivity comparison of IS*6110-*dPCR (cut-off value = 2.6 copies/20-μL reaction mixture) between definite and probable pleural TB (*P* = 0.000, Pearson chi-square test).

c Sensitivity comparison of IS*1081*-dPCR (cut-off value = 2.1 copies/20-μL reaction mixture) between definite and probable pleural TB (*P* = 0.000, Pearson chi-square test).

d AUC, area under receiver operating characteristic (ROC) curve.

e LR+, positive likelihood ratio. -, not applicable.

f LR−, negative likelihood ratio.

g PPV, positive predictive value.

h NPV, negative predictive value.

i CI, confidence interval.

### Sensitivity comparison of PF dPCR assay with other tests.

The positive detection rates of different tests in patients with pleural TB are listed in Table 3. The sensitivity of PF IS*6110* OR IS*1081*-dPCR was significantly higher than that of etiological tests, including smear microscopy (57.4% versus 7.1%), mycobacterial culture (55.3% versus 31.8%), and Xpert (57.6% versus 23.0%) (all *P* values < 0.001). Compared with immunological tests, the sensitivity of PF IS*6110* OR IS*1081*-dPCR was significantly lower than that of peripheral blood T-SPOT.TB (58.8% versus 91.9%, *P* < 0.001), but higher than that of TB antibody detection in peripheral blood and TB antibody detection in PF (60.7% versus 42.9%, *P* = 0.002; 71.5% versus 40.4%, *P* = 0.002). There was no significant difference between PF IS*6110* OR IS*1081*-dPCR with pleural biopsy in conjunction with acid-fast bacilli test (58.8% versus 47.1%, *P* = 0.727) or PF ADA test when cut-off value was 40 U/L (64.2% versus 70.1%, *P* = 0.358).

**TABLE 3:** Sensitivity comparisons of PF dPCR assay with diagnostic tests in diagnosis of pleural TB

Tests	No. of patients	Sensitivity	*P* value
PF IS*6110* OR IS*1081*-dPCR^*a*^ compared with routine etiological tests			
PF^*c*^ IS*6110* OR IS*1081*-dPCR vs PF smear microscopy	155	57.4% (89/155) vs 7.1% (11/155)	0.000
PF IS*6110* OR IS*1081*-dPCR vs PF mycobacterial culture	132	55.3% (73/132) vs 31.8% (42/132)	0.000
PF IS*6110* OR IS*1081*-dPCR vs PF Xpert MTB/RIF	165	57.6% (95/165) vs 23.0% (38/165)	0.000
PF IS*6110* OR IS*1081*-dPCR compared with immunological tests
PF IS*6110* OR IS*1081*-dPCR vs peripheral blood IGRAs^*d*^ (T-SPOT.TB)^*b*^	154	59.1% (91/154) vs 92.9% (143/154)	0.000
PF IS*6110* OR IS*1081*-dPCR vs TB antibody detection in peripheral blood	168	60.7% (102/168) vs 42.9% (72/168)	0.002
PF IS*6110* OR IS*1081*-dPCR vs TB antibody detection in PF	151	71.5% (108/151) vs 40.4% (61/151)	0.002
PF IS*6110* OR IS*1081*-dPCR compared with pleural biopsy
PF IS*6110* OR IS*1081*-dPCR vs pleural biopsy in conjunction with acid-fast bacilli test	16	58.8% (10/17) vs 47.1% (8/17)	0.727
PF IS*6110* OR IS*1081*-dPCR compared with biochemical parameters
PF IS*6110* OR IS*1081*-dPCR vs PF ADA^*e*^ (when cut-off value is 40 U/L)	137	64.2% (88/137) vs 70.1% (96/137)	0.358

a The cut-off values of PF IS*6110* OR IS*1081-dPCR* assay were 2.6 and 2.1 copies/20-μL reaction mixture, respectively. One of them was positive meant the dPCR result was positive. McNemar test was used to determine the significance between PF IS*6110* OR IS*1081-dPCR* assay and other tests.

b The diagnostic criteria of peripheral blood T-SPOT.TB was recommended by the manufacturer.

c PF, pleural fluid.

d IGRAs, interferon-gamma release assays.

e ADA, adenosine deaminase.

### Risk factors for negative PF dPCR results.

Multivariate logistic regression analysis revealed that long antituberculosis treatment time (>1 month) was the independent risk factor for negative dPCR results (odds radio [OR] = 3.541, *P* = 0.025, Table 4). The number of targets detected and the positive rate of dPCR in treatment group (≤1 month) were both higher than those in treatment group (>1 month) (IS*6110,* *P* = 0.001, IS*1081, P* = 0.006 in Fig. S2; 61.7% [103/167] versus 31.3% [5/16] in Table 4).

**TABLE 4:** Univariate and multivariate analysis of risk factors associated with negative dPCR results in pleural TB patients

Characteristics	dPCR		Univariate analysis	Multivariate analysis	
	Negative	Positive	*P* value	Odds ratio (95% CI)	*P* value
Age, median (range), yr	48 (16 to 91)	47 (17 to 93)	0.933		
Gender (male/female)	62/13	87/21	0.718		
Duration of illness before hospitalization			0.032		
≤1 mo	31/75 (41.3%)	62/108 (57.4%)			
1 to 6 mo	25/75 (33.3%)	28/108 (25.9%)			
> 6 mo	19/75 (25.3%)	18/108 (16.7%)			
Antituberculosis treatment time			0.018		
≤1 mo	64/75 (85.3%)	103/108 (95.4%)			
>1 mo	11/75 (14.7%)	5/108 (4.6%)		3.541 (1.176 to 10.660)	0.025
Effusion site			0.094		
Left	31/75 (41.3%)	62/108 (57.4%)			
Right	25/75 (33.3%)	28/108 (25.9%)			
Bilateral	19/75 (25.3%)	18/108 (16.7%)			
Underlying diseases					
Diabetes mellitus	11/75 (14.7%)	14/108 (13.0%)	0.741		
HIV-positive	1/72 (1.39%)	0/106 (0.0%)	0.404		
Liver diseases	30/75 (40.0%)	31/106 (29.2%)	0.132		
Syphilis	2/72 (2.8%)	3/105 (2.9%)	1.000		
Hypertension	8/75 (10.7%)	18/108 (16.7%)	0.253		

## DISCUSSION

The great limitations of existing assays for diagnosis of pleural TB make it urgent to develop more efficient methods. Our study for the first time comprehensively evaluates the diagnostic accuracy of PF digital PCR analysis for pleural TB. The results showed that the sensitivity of PF IS*6110* OR IS*1081-*dPCR for total, definite, and probable pleural TB was 59.0% (95% CI = 51.5% to 66.2%), 72.8% (95% CI = 62.6% to 81.6%), and 45.1% (95% CI = 34.6% to 55.8%), respectively, while the specificity was 100% (95% CI = 97.1% to 100.0%). It indicates that PF IS*6110* OR IS*1081-*dPCR is an efficient method for pleural TB diagnosis and is more sensitive than Xpert, an important test recommended by the World Health Organization (6, 10–12).

M. tuberculosis-specific targets are crucial for dPCR analysis. It is known that there are 16 copies of IS*6110* and six copies of IS*1081* within the genome of M. tuberculosis H37Rv strain, and IS*6110* is commonly used as a target for detecting the presence of M. tuberculosis (25, 31–34). However, a minority of M. tuberculosis isolates carry a single or zero copy of IS*6110* (35–38). Therefore, we selected IS*6110* and IS*1081* as the detection targets in this study. The combination of two targets can theoretically reduce false-negative results and improve the sensitivity of detection (39, 40). Our experimental results showed that four of the 183 pleural TB patients were IS*6110*-dPCR negative and IS*1081*-dPCR positive, which supports the necessity of using these two targets for dPCR detection. In addition, we used UNG and dUTP (instead of dTTP) in the dPCR reaction to effectively control carry-over from previous PCR products, which is very meaningful to prevent contamination and avoid false-positive results in clinical practice (41).

In this study, a very small amount of M. tuberculosis DNA can be detected in several non-TB samples. This could not be the cause of contamination, as no M. tuberculosis DNA was detected in all negative controls randomly distributed. Similarly, some other studies have also observed M. tuberculosis genes or peptides in serum exosomes derived from healthy or latent tuberculosis infection (LTBI) people (42, 43). Traces of circulating M. tuberculosis DNA can also be detected in the plasma of some health care workers who have been confirmed not to have previous M. tuberculosis infection by T-SPOT.TB (31) or LTBI persons (44). Why a small amount of M. tuberculosis nucleic acid is present in the body fluid of non-TB people is not fully understood. This may be related to the fact that people have been infected with M. tuberculosis, which has been eliminated or is in a latent infection state. Therefore, the final result of dPCR assay for disease diagnosis is determined by the cut-off value of targets rather than by the presence of positive droplets or targets, just as the cut-off value of Ct needs to be set in qPCR. It should be noted that the cut-off value needs to be carefully set according to the test data of the disease group and the control group with a large sample size. For clinical samples with copy number significantly higher than the high detection limit, accurate quantitative results will not be obtained according to the Poisson distribution, but the samples will be given very large values, which will be judged as the positive results, because any value greater than the cut-off is considered positive. In fact, such samples are relatively rare in tuberculous PF samples due to the paucibacillary nature. In this study, 183 pleural TB patients were tested and the highest quantitative dPCR results (IS*6110*, 9990 copies/20 μL-reaction mixture; IS*1081*, 2020 copies/20-μL reaction mixture) were within the linear ranges.

We also compared the sensitivity of PF IS*6110* OR IS*1081-*dPCR with routine diagnostic tests. It had higher sensitivity than PF smear (8.1-fold), mycobacterial culture (1.7-fold), and Xpert MTB/RIF (2.5-fold), indicating that it was more sensitive than current bacteriological and molecular tests which can provide evidence of M. tuberculosis or its nucleic acids in PF. It has been reported that ultrasound, computed tomography (CT), or thoracoscopy-guided pleural biopsy can improve the diagnosis of pleural TB (15, 45, 46). In this study, we found no significant difference in sensitivity between PF IS*6110* OR IS*1081*-dPCR and pleural biopsy in conjunction with acid-fast bacilli. However, we think that pleural biopsy combined with molecular biological tests such as digital PCR and Xpert can greatly improve the diagnosis of pleural TB (14, 15, 46). Compared with immunological tests, the sensitivity of PF IS*6110* OR IS*1081-*dPCR was significantly lower than that of peripheral blood IGRAs (58.8% versus 91.9%, *P* < 0.001) and higher than that of TB antibody detection in peripheral blood (60.7% versus 42.9%, *P* = 0.002) or in PF (71.5% versus 40.4%, *P* = 0.002). It is worth noting that the diagnostic accuracy of IGRAs is unsatisfactory and heterogeneous in different studies (the pooled sensitivity and specificity of blood IGRAs were 0.77 [95% CI = 0.71 to 0.83] and 0.71 [95% CI = 0.65 to 0.76], and those of PF IGRAs were 0.72 [95% CI = 0.55 to 0.84] and 0.78 [95% CI = 0.65 to 0.87] in a meta-analysis) (18, 47–49). We also found that there was no correlation between the copy number of digital PCR (IS*6110* or IS*1081*) and the IFN-γ spot-forming cells (SFC) number (ESAT-6 or CFP-10) from peripheral blood or pleural fluid samples (all |*r*|<0.1, all *P* > 0.2). Moreover, there were no significant differences in sensitivity between PF IS*6110* OR IS*1081*-dPCR and PF ADA (when cut-off value is 40 U/L). ADA is a purine-degrading enzyme found in many types of cells, especially in active T-cells. High ADA levels can also be observed in patients with empyema, malignancy, para-pneumonic effusions, rheumatoid pleurisy, and some other infectious diseases such as brucellosis (2, 13, 50). Therefore, considering their poor specificity, PF IS*6110* OR IS*1081*-dPCR has obvious advantages over current immunological tests and biochemical indicators in diagnosing pleural TB.

Risk factors for negative PF IS*6110* OR IS*1081*-dPCR results are also noteworthy. Among the factors, including age, gender, duration of illness before hospitalization, antituberculosis treatment time, effusion site, and underlying diseases, long antituberculosis treatment time (>1 month) was found to be significantly associated with negative dPCR results in pleural TB patients. The copy number of targets detected and the positive rate of dPCR in antituberculosis treatment group (>1 month) were both significantly lower than those in treatment group (≤1 month). These results suggest that the copy number of nucleic acids detected by PF IS*6110* OR IS*1081*-dPCR may decrease with the increase of antituberculosis treatment time, which reminds us of the importance of early PF dPCR detection. However, whether it can be used to monitor the therapeutic effect needs further evaluation.

This study had some limitations. First, we used PF samples frozen at −80°C over a period of 8 months to 4 years for DNA extraction and digital PCR detection. A previous study found a slightly increased Ct value when using frozen samples compared with fresh ones by Xpert (51). Second, we used a common and convenient method to extract total DNA from 700-μL PF. It is reported that enrichment of cell-free DNA from large volume PF can improve the sensitivity of qPCR assay for pleural TB (52, 53). More studies using samples under various storage conditions or optimizing PF DNA extraction methods are needed to fully understand the diagnostic value of dPCR for pleural TB.

In conclusion, PF IS*6110* OR IS*1081*-dPCR was shown to be an accurate molecular assay which can provide direct etiological evidence. It is more sensitive than current microbiological and commercial molecular tests, and has considerable potential in improving the diagnosis of pleural TB.

## MATERIALS AND METHODS

### Study participants.

In-patients aged ≥ 16 years with evidence of pleural effusion, suspected to have pleural TB, were consecutively enrolled from 2015 to 2019 at Beijing Chest Hospital, Beijing, China. Some of the following tests were performed to help make a final diagnosis: MRI, CT, and ultrasonic examinations; PF tests related to TB, including smear microscopy for acid-fast bacilli with 50-μL PF (direct smear with Auramine staining), mycobacterial culture with 0.5 mL PF using Bactec MGIT 960 system (Becton, Dickinson and Company, Franklin Lakes, NJ, USA), Xpert with 1 mL PF and M. tuberculosis antibody detection using purified specific antigen components of H37Rv strain (Huian, Shenzhen, Guangdong, China); blood tests related to TB, including IGRAs (T-SPOT.TB; Oxford Immunotec Ltd., Abingdon, UK) and M. tuberculosis antibody detection; biochemical, cytologic, and histopathological examination of PF (6, 47, 54). All of the commercial assays were performed as per the manufacturer’s instructions. The study was approved by the Ethics Committee of Beijing Chest Hospital, Capital Medical University.

### Categorization of patients.

Patients were divided into three groups according to the diagnostic criteria (6, 55): (i) definite pleural TB: with positive imaging findings and positive etiological test results for PF using any of the smear microscopy, culture, or commercial NAAT for M. tuberculosis; or with positive imaging findings and positive pathological findings in PF or pleural biopsy tissue; (ii) probable or clinically diagnosed pleural TB: with negative PF etiological test results and positive imaging findings, exudative pleural effusion, elevated PF adenosine deaminase, and positive immunological results (TST or IGRAs or M. tuberculosis antibody); (iii) non-TB: an alternative diagnosis was made and all tests were not suggestive of TB.

### Pleural fluid collection and DNA extraction.

A total of 30 to 50 mL of PF per patient were collected and sent to the laboratory for the above routine clinical tests. The remaining PF were centrifuged at 2,000 g for 10 min at room temperature and the supernatants were frozen in aliquots at −80°C. Then, 700 μL PF was used to extract DNA in batches using DNeasy Blood and Tissue Kits (69506, Qiagen, Hilden, Germany) with an elution volume of 50 μL. The DNA samples were stored at −80°C until dPCR detection.

### Digital PCR analysis.

IS*6110* and IS*1081* were both conserved DNA sequences in M. tuberculosis complex and were used as detection targets in this study. The primers for amplification and the internal oligonucleotide probes: IS*6110*-forward, 5′-GGCGTACTCGACCTGAAAGA-3′, IS*6110*-reverse, 5′-CTGAACCGGATCGATGTGTA-3′, IS*6110*-probe, 5′-(FAM)-CCACCATACGGATAGGGGAT-(BHQ-1)-3′, IS*1081*-forward, 5′-CCTGCTGCACTCCATCTAC-3′, IS*1081*-reverse, 5′-CGTCGAGTACCCGATCATAT-3′, IS*1081*-probe, 5′-(HEX)-CCCGACGCCGAATCAGTTGT-(BHQ-2)-3′ (31, 39, 56). All the primers and probes were synthesized by Sangon Biotech (Shanghai, China). Twenty μL of the reaction mixture contained 10-μL ddPCR supermix for probes (1863010, Bio-Rad, Hercules, CA, USA), 0.9 μM each primer, 0.2 μM each probe, 0.3 U uracil-N-glycosylase (UNG), and 5.3 μL extracted DNA without dilution. The entire mixtures and 70 μL of droplet generation oil were added in cartridges and loaded into a QX200 droplet generator (Bio-Rad) for droplet generation. The droplet emulsions were transferred to a 96-well plate and sealed with a foil heat seal. PCR condition: 37°C for 10 min, 95°C for 10 min, 40 cycles of 94°C for 30 s and 54°C for 40 s, 98°C for 10 min. The temperature ramp rate was 2.0°C/s. After amplification, the plate was loaded on a QX200 droplet reader (Bio-Rad) to acquire the fluorescence signal of each droplet. Data analysis was performed using QuantaSoft Version 1.7.4.0917 (Bio-Rad). The threshold was manually set between the negative and positive droplet clusters of the control samples based on the fluorescence amplitudes, which was used to discriminate positive droplets from negative droplets. The absolute quantities of target DNA in reaction were automatically calculated based on the Poisson distribution. No-template negative control and M. tuberculosis DNA positive control were adopted in each assay. Each sample was tested in two independent experiments (each led in duplicate) and the number of copies per 20-μL reaction mixture was calculated as the average of two independent experiments results.

### Statistical analysis.

Statistical analysis was performed using SPSS Version 13.0 (SPSS, Chicago, IL, USA). Categorical variables were tested by Chi-square test or McNemar test, while continuous variables were compared by the Student's *t* test, Mann-Whitney *U* test, or Wilcoxon test, as appropriate. The independent risk factors were analyzed using a multivariate logistic regression model with forward stepwise (likelihood ratio) selection. Coefficient of determination (R^2^) of quantification was assessed for both IS*6110* and IS*1081* by linear regression analysis by plotting the measured copies of the standards and comparing them with expected values of serial dilutions. The CV was calculated as the standard deviation divided by replicate mean. Accordance of different assays was analyzed by Spearman correlation test. Two-sided *P* < 0.05 were considered significant.
