# Supplementary material for: Use of Pleural Fluid Digital PCR Analysis to Improve the Diagnosis of Pleural Tuberculosis
Source: Microbiol Spectr. 2022 Oct 20;10(6):e01632-22. doi: 10.1128/spectrum.01632-22 (PMC9769588; doi:10.1128/spectrum.01632-22)
Supplement: Supplemental file 1 — Fig. S1 and S2. Download spectrum.01632-22-s0001.pdf, PDF file, 0.2 MB [file spectrum.01632-22-s0001.pdf]

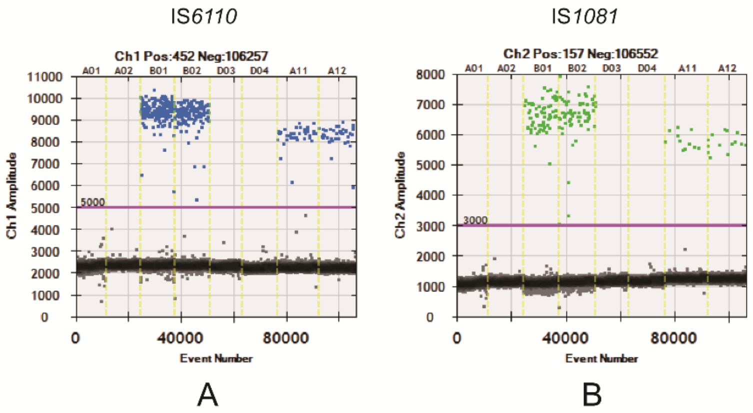

Fig. S1. Measurement of IS*6110* and IS*1081* in pleural fluid DNA samples by droplet dPCR analysis. (A) Part of the original droplets pictures of IS*6110*-dPCR. (B) Part of the original droplets pictures of IS*1081*-dPCR. A01 and A02, negative controls; B01 and B02, positive controls; D03 and D04, samples from a non-TB patient; A11 and A12, samples from a pleural TB patient.

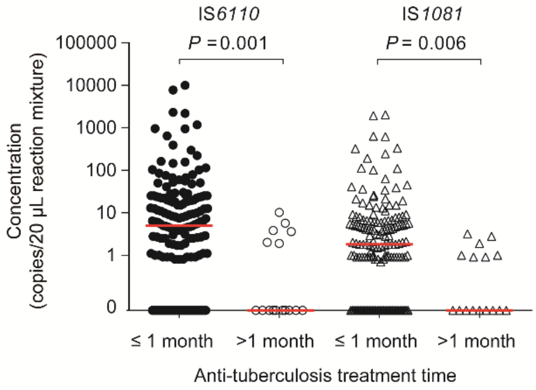

Fig. S2. Differences in the number of *M. tuberculosis* IS*6110* and IS*1081* copies detected in pleural fluid DNA samples between the two groups after anti-tuberculosis treatment (≤ 1 month group and > 1 month group).
